# Supplementary material for: Malnutrition disrupts adaptive immunity during visceral leishmaniasis by enhancing IL-10 production
Source: PLoS Pathog. 2024 Nov 11;20(11):e1012716. doi: 10.1371/journal.ppat.1012716 (PMC11581394; doi:10.1371/journal.ppat.1012716)
Supplement: S1 Fig — The percentage of IL-10 in cells from the spleen and liver of naive or L. infantum-infected control diet or polynutrient deficient diet (PND diet) at 6 weeks of diet and 4 weeks post-infection. Leukocytes from the spleen and liver were stimulated with PMA, ionomycin, and monensin in the presence of BFA for 2.5 hr and subsequently harvested and stained for IL-10. Cells were gated based on size (FSC) and granularity (SSC), singlet cells, live+, and CD45+ cells. For NK and NKT cell identification, the NK1.1+ subset was identified as CD3-NK1.1+ and CD3+NK1.1+, respectively. NK1.1- subset was gated on CD3+ subset for subsequent identification of CD4+IFNγ+, CD4+ Foxp3+, and CD8+ cells. CD3- subset was followed by gate on CD19+ subset for B cell. For myeloid population identification, the CD19- subset was followed by the gate on CD11c+Ly6C- (dendritic cells), CD11c-Ly6C+CD11b+ (monocytes), and CD11c-Ly6C-F4/80+ (macrophages). The statistical significance was calculated by one-way ANOVA (*p < 0.05, **p < 0.01, and ***p < 0.001). (DOCX) [file ppat.1012716.s001.docx]

**S1 Fig. IL-10 production by lymphoid and myeloid populations in malnourished mice.** The percentage of IL-10 in cells from the spleen and liver of naive or *L. infantum*-infected control diet or polynutrient deficient diet (PND diet) at 6 weeks of diet and 4 weeks post-infection. Leukocytes from the spleen and liver were stimulated with PMA, ionomycin, and monensin in the presence of BFA for 2.5 hr and subsequently harvested and stained for IL-10. Cells were gated based on size (FSC) and granularity (SSC), singlet cells, live^+^, and CD45^+^ cells. For NK and NKT cell identification, the NK1.1^+^ subset was identified as CD3^-^NK1.1^+^ and CD3^+^NK1.1^+^, respectively. NK1.1^-^ subset was gated on CD3^+^ subset for subsequent identification of CD4^+^IFNγ^+^, CD4^+^ Foxp3^+^, and CD8^+^ cells. CD3^-^ subset was followed by gate on CD19^+^ subset for B cell. For myeloid population identification, the CD19^-^ subset was followed by the gate on CD11c^+^Ly6C^-^ (dendritic cells), CD11c^-^Ly6C^+^CD11b^+^ (monocytes), and CD11c^-^Ly6C^-^F4/80^+^ (macrophages). The statistical significance was calculated by one-way ANOVA (*p < 0.05, **p < 0.01, and ***p < 0.001).
