# Supplementary material for: Malnutrition disrupts adaptive immunity during visceral leishmaniasis by enhancing IL-10 production
Source: PLoS Pathog. 2024 Nov 11;20(11):e1012716. doi: 10.1371/journal.ppat.1012716 (PMC11581394; doi:10.1371/journal.ppat.1012716)
Supplement: S2 Fig — Levels of IL-1β, IL-6, and TNFα in supernatants from a liver homogenate at 6 weeks of diet and 4 weeks post-infection. The data are expressed as mean ± SEM and represent the combination of two experiments. The statistical significance was calculated by one-way ANOVA (*p < 0.05, **p < 0.01, and ***p < 0.001). (DOCX) [file ppat.1012716.s002.docx]

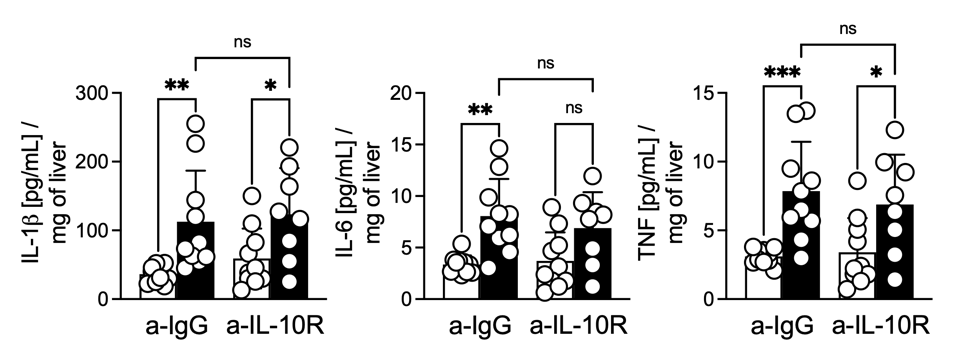


**S2 Fig. IL-10R blockade has no impact on systemic inflammation.** Levels of IL-1β, IL-6, and TNFα in supernatants from a liver homogenate at 6 weeks of diet and 4 weeks post-infection. The data are expressed as mean ± SEM and represent the combination of two experiments. The statistical significance was calculated by one-way ANOVA (*p < 0.05, **p < 0.01, and ***p < 0.001).
