# Supplementary material for: Malnutrition disrupts adaptive immunity during visceral leishmaniasis by enhancing IL-10 production
Source: PLoS Pathog. 2024 Nov 11;20(11):e1012716. doi: 10.1371/journal.ppat.1012716 (PMC11581394; doi:10.1371/journal.ppat.1012716)
Supplement: S1 Supporting_Protocol — (DOCX) [file ppat.1012716.s003.docx]

**Supporting protocol**

*Spleen cells culture*

Single-cell suspensions of spleen samples were aseptically prepared, diluted to a concentration of 2 x 16^6^ cells/mL, and dispensed into 48-well plates in total volume of 500 μL of complete PRMI-1640 medium, and stimulated with or without *L. infantum* crude antigen for 72h of culture at 37°C in 5% CO_2_.

*Quantification of cytokines*

The concentrations of IL-10 in the supernatants of restimulated splenocytes were determined using an IL-10 ELISA kit. To detect IL-10, IL-1β, IL-6, and TNF-α in the liver, total organ and tissue fragments were weighed and titrated in 1 mL of Complete EDTA-free Protease Inhibitor Cocktail. The levels of cytokines were determined using commercial ELISA kits.

*Intrahepatic cells isolation*

Intrahepatic inflammatory cells were obtained by incubating in collagenase (200 μg/mL) and Deoxyribonuclease I (10 μg/mL) for 30 min at 37°C, homogenizing, and subjecting to a 40% Percoll gradient.

*Cell culture, stimulation and flow cytometry*

For intracellular staining, single-cell suspensions were incubated with PMA (50 ng/mL), ionomycin (500 ng/mL), monensin (1000x, eBioscience), and Brefeldin A (10 μg/mL) for 4h. Cells were stained with LIVE/DEAD Fixable Aqua Dead Cell Stain Kit and subsequently incubated with anti-CD16/CD32 and 10% rat-IgG1. For surface staining, cells were incubated with monoclonal anti-CD45, anti-CD3, anti-CD4, anti-CD8, anti-NK1.1, anti-CD19, anti-CD11c, anti-Ly6C, anti-CD11b, and anti-F4/80. For intracellular staining, cells were permeabilized with a Foxp3/Transcription Factor staining buffer set according to the manufacturer’s guide and stained with anti-Foxp3, anti-IL-10, anti-T-bet, and anti-IFNγ. The total leukocyte counts were determined by measuring the relative expression of the leukocyte subpopulations stained with a specific antibody within a set of 300,000 acquired events as a proportion of the total leukocyte number per organ obtained in a Neubauer chamber. Data were collected using LSRIII Fortessa/Symphony A3 and analyzed using FlowJo.

Resources table

| **Reagent** | **Source** | **Cat number** |
| --- | --- | --- |
| APCcy7 anti-mouse CD45, clone 30-F11 | Biolegend | Cat #103116 |
| BV605 anti-mouse CD3, clone 172A | Biolegend | Cat #100237 |
| BV650 anti-mouse CD4, clone RM4-5 | Biolegend | Cat #100545 |
| BUV395 anti-mouse CD3, clone 145-2C11 | BD Biosciences | Cat #563565 |
| BUV805 anti-mouse CD4, clone RM4-5 | BD Biosciences | Cat #741912 |
| Percp-cy5.5 anti-mouse CD8, clone 53-6.7 | BD Biosciences | Cat #551162 |
| BV711 anti-mouse NK1.1, clone PK136 | Biolegend | Cat #108745 |
| AF647 anti-mouse T-bet, clone 4B10 | eBioscience | Cat #51-5825-80 |
| PEcy7 anti-mouse IFNγ, clone XMG1.2 | Thermo Fisher Scientific | Cat #25-7311-82 |
| APC anti-mouse Foxp3, clone FJK-16S | Thermo Fisher Scientific | Cat #7-5773-80 |
| eF450 anti-mouse CD11b, clone M1/70 | eBioscience | Cat #48-0112-82 |
| BUV661 anti-mouse CD11b, clone M1/70 | BD Biosciences | Cat #612977 |
| FITC anti-mouse CD11c, clone N418 | eBioscience | Cat #11-0114-82 |
| BUV785 anti-mouse Ly6C, clone HK1.4 | Biolegend | Cat #128041 |
| BV605 anti-mouse F4/80, clone BM8 | Biolegend | Cat #123133 |
| PE anti-mouse iNOS, clone CXNFT | Thermo Fisher Scientific | Cat #12-5920-82 |
| PE anti-mouse iNOS, clone CXNFT | Thermo Fisher Scientific | Cat #12-5920-82 |
| AF647 anti-mouse T-bet, clone 4B10 | eBioscience | Cat #51-5825-80 |
| PEcy7 anti-mouse IFNγ, clone XMG1.2 | Thermo Fisher Scientific | Cat #25-7311-82 |
| LIVE/DEAD Fixable Aqua Dead Cell Stain Kit | Molecular Probes | Cat #L34957 |
| CD16/CD32 Monoclonal Antibody, clone 93 | eBioscience | Cat #14-0161-86 |
| IgG1 from rat serum | Sigma-Aldrich | Cat #I8015 |
| ACK lysing buffer |  | Cat #118-156-101 |
| anti-mouse IL-10R antibody, clone 1B1.3A | BioXCell | Cat #BE0050 |
| anti-IgG1 isotype control, clone HRPN | BioXCell | Cat #BE0088 |
| Complete EDTA-free Protease Inhibitor Cocktail | Sigma | Cat # 11836170001 |
| BD Cytofix/Cytoperm | BD Biosciences | Cat #554714 |
| Foxp3 / Transcription Factor Staining Buffer Set | Thermo Fisher Scientific | Cat # 00-5523-00 |
| Mouse IL-10 ELISA Kit | R&D Systems | Cat #M1000B |
| Mouse IL-1β ELISA Kit | Biolegend | Cat #432601 |
| Mouse IL-6 ELISA Kit | Biolegend | Cat #431301 |
| Mouse TNFα ELISA Kit | Biolegend | Cat #430901 |
