# Supplementary material for: Malnutrition disrupts adaptive immunity during visceral leishmaniasis by enhancing IL-10 production
Source: PLoS Pathog. 2024 Nov 11;20(11):e1012716. doi: 10.1371/journal.ppat.1012716 (PMC11581394; doi:10.1371/journal.ppat.1012716)
Supplement: Attachment — Submitted filename: Response to Reviewers.docx [file ppat.1012716.s004.docx]

Phillip Scott, PhD

Professor of Immunology

Vice Dean *for* Research & Academic Resources

University of Pennsylvania

School of Veterinary Medicine

October 29, 2024

To the Editors of *Plos Pathogens*:

We are resubmitting our manuscript entitled “**Malnutrition disrupts adaptive immunity during visceral leishmaniasis by enhancing IL-10 production**”. We appreciate the comments by the reviewers and have addressed all the comments raised by the reviewers. The point-by-point responses to the reviewer’s comments are below.

Thank you for your interest in our work.

Sincerely,

Phillip Scott, Ph.D.

Professor of Immunology

Corresponding Author

380 South University Ave.

University of Pennsylvania

Philadelphia, PA 19104

email: [pscott@upenn.edu](mailto:pscott@upenn.edu)

phone: 215-898-1602

fax: 215-747-2294

**Title: Malnutrition disrupts adaptive immunity during visceral leishmaniasis by enhancing IL-10 production**

**Reviewer's Responses to Questions**

**Part I - Summary**

 Reviewer #1: Sacramento et al., use a poly-nutrient deficient mouse model that mimics the most commonly observed malnutrition to study the immune mechanism of L. infantum infection mediated pathogenesis. Previous studies using the PND mouse models reported deficient innate immune responses, but the impact on adaptive immunity remained unexplored. In this study the authors report elevated IL-10 levels under the PND regimen and that blocking IL-10 controlled the splenic parasite burden. Overall the studies are well conducted and the conclusions are robust.

Reviewer #2: The manuscript by Sacramento, et al, describes the outcome of chronic systemic Leishmania infantum infection of malnourished vs. control mice. It identifies increased IL-10 production as a driver of the increased severity of L. infantum infection in malnourished mice because antibody-mediated blockade of the IL-10 receptor improved IFNg production by CD4 T cells, hepatic granuloma formation, and parasite control. The experimental design and execution are excellent and the manuscript is well-written. While the results are interesting and add to our understanding of the impact of malnutrition on adaptive anti-Leishmania immunity, the data stop short of establishing a clear mechanistic understanding of the contribution of malnutrition to the immunopathogenesis of visceral leishmaniasis.

**Part II – Major Issues: Key Experiments Required for Acceptance**
Please use this section to detail the key new experiments or modifications of existing experiments that should be absolutely required to validate study conclusions.

Generally, there should be no more than 3 such required experiments or major modifications for a "Major Revision" recommendation. If more than 3 experiments are necessary to validate the study conclusions, then you are encouraged to recommend "Reject".

Reviewer #1: (No Response)

Reviewer #2:

1. The source of IL-10 is critical to understanding how malnutrition influences the immunopathogenesis of visceral leishmaniasis. Is it driven by polarized or mixed-phenotype CD4 T cells or part of a pathologic innate immune response as has been previously reported in this model? It would be relatively straightforward to determine the cellular source of the IL-10 (CD4 T cells or innate immune cells) by intracellular staining, as was done for IFNg and iNOS.
***Answer:*** The flow cytometry analysis demonstrates that IL-10 is produced by many cells during VL, especially by Th1 cells (Tr1), as previously reported (Bunn et al., 2018; Edwards et al., 2023). Interestingly, malnutrition enhances the frequency of NK cells and monocytes producing IL-10. Please see lines 103-106 and Supporting Figure 1.

2. Since myeloid cells expressing iNOS were reduced in PND mice, it would be helpful to determine if this was a direct autocrine/paracrine effect of IL-10, impaired IFNg responsiveness of myeloid cells in PND, or indirect through suppression of T cell IFNg production. This could be teased out with ex vivo cell culture experiments.
***Answer:*** The reviewer is correct that IL-10 can block iNOS in several ways. However, we felt that the ex-vivo experiments suggested would be a bit tricky and the results difficult to interpret, since it would be challenging to establish the appropriate conditions that would mimic the malnourished in vivo conditions, particularly the appropriate culture media to use for the experiments. Since the blockade of IL-10 led to an increase in IFNγ production, we hypothesize this increase is the most likely explanation for the increase in iNOS. To respond to the reviewer’s comment, however, we have added in the text that there are other non-mutually exclusive possibilities for how blockade of IL-10 restores iNOS (Please see lines 159-161).

3. Since exaggerated systemic inflammation in this model seems to be the driver of growth faltering (reference 30), and parasite dissemination from the skin (doi.org/10.1371/journal.pntd.0011040), and the increased IL-10 could be an innate regulatory response to control the inflammation, it is surprising that IL-10R blockade did not worsen the growth metrics. Perhaps this supports the notion that IL-10R blockade is primarily targeting the T cell anti-Leishmania adaptive response. As noted above, ex vivo and in vitro studies could help sort this out. Although it is not the focus of this manuscript, it would be interesting to know how IL-10R blockade affected systemic inflammatory markers.

***Answer:*** We measured cytokine levels in liver samples to investigate whether IL-10R blockade affects systemic inflammatory markers. Corroborating the previous report, malnourished mice have increased levels of IL-1β, IL-6, and TNF-α compared with control mice at baseline (Patterson et al., 2022), and the α-IL-10R mAb treatment has no impact on the modulation of systemic cytokine levels. Please see lines 142-146 and Supporting Figure 2.

**Part III – Minor Issues: Editorial and Data Presentation Modifications**
Please use this section for editorial suggestions as well as relatively minor modifications of existing data that would enhance clarity.

Reviewer #1: The data presented in the manuscript supports the hypothesis that the main mechanism of VL pathogenesis in a malnutrition condition to be IL-10 mediated. Blocking of IL-10 did not alter the number of CD4+ T cells but improved the IFN-g producing CD4+ T cells along with iNOS+ cells. Accordingly, liver and spleen parasite burdens were controlled upon IL-10 blocking treatment of L. infantum infected PND mice.

While the manuscript shows the correlation between malnutrition and an IL-10 mediated anti-inflammatory response, which was also observed in sepsis and fungal infections, a mechanistic understanding is currently lacking. Several bacterial/fungal infection studies reported the identification of IDO1-AhR signaling mediated IL-10 production. The authors may expand the discussion section on potential metabolic immune regulation that may underlie the elevated IL-10 expression. Please see https://doi.org/10.1007/s00394-018-1679-0 for an excellent coverage.
***Answer:*** We appreciate the suggestion. Malnutrition disturbs circulating metabolite levels, enhancing tryptophan catabolites such as kynurenine (Mayneris-Perxachs et al., 2016). Given the influence of tryptophan catabolism on immunosuppression, the high level of IL-10 might result from kynurenine axis stimulation (Seo and Kwon et al., 2023; Lanis ey al., 2017). We have added a comment to that effect in the text (please see lines 173-176). We have not added a lengthier discussion due to limits on the manuscript size.

Have the authors considered a rescue group where normal diet is provided to the PND mice after the L infantum infection to verify if IL-10 levels alter in sync with the nutrition?

***Answer:*** In future studies, we consider rescuing the diet to evaluate IL-10 levels, anti-parasitic mechanisms, and the impact on the systemic inflammatory response. A recently published study demonstrated that refeeding chronically malnourished mice recovers body mass and cellularity across immune organs and T cell response. However, refed mice remain susceptible to Listeria infection due to a defective emergency myelopoiesis and deficient neutrophil and monocyte peripheral response (Sukhina et al., 2024), suggesting that malnutrition impacts different protective immune mechanisms.

Reviewer #2: 4. The authors should recognize the work of the Cuervo group (DOI:10.1371/journal.pone.0114584) who previously showed increased parasite load, increased IL-10 and reduced IFNg production in the spleen of L. infantum infected protein-deficient mice.

***Answer:*** We cited the work on the new version of the manuscript.

5. It would be helpful to label the tissue sources in the different panels in Figs. 1 and 2.
***Answer:*** We appreciate the suggestion. Please see the new figures.

6. Reference 11 does not seem to fit with where it is cited.

***Answer:*** The review is correct. The reference was removed.
